# Clinical long-term outcome of hepatitis D compared to hepatitis B monoinfection

**DOI:** 10.1007/s12072-023-10575-0

**Published:** 2023-10-03

**Authors:** Anika Wranke, Benjamin Heidrich, Katja Deterding, Katharina Luise Hupa-Breier, Janina Kirschner, Birgit Bremer, Markus Cornberg, Heiner Wedemeyer

**Affiliations:** 1 Department of Gastroenterology, Hepatology and Endocrinology, Hannover Medical School, Carl-Neuberg-Straße 1, 30625 Hannover, Germany; 2 German Center for Infection Research (DZIF), Partner Sites: Hannover - Braunschweig, Germany; 3 D-Solve Consortium, Hannover, Germany; 4 Excellence Cluster Resist, Hannover, Germany

**Keywords:** Hepatitis delta, Hepatitis B, Therapy, Hepatocellular carcinoma, Liver decompensation, Cirrhosis, Clinical longterm outcome, HBeAg, Nucleos(t)ide analogs, Pegylated interferon alpha

## Abstract

**Background and aims:**

Hepatitis D virus (HDV) infection causes the most severe form of chronic viral hepatitis. However, it is still unclear to what extent the underlying cirrhosis may contribute to disease progression. The aim of this study was to compare the long-term outcome of HDV infection with HBV monoinfection in a single-center cohort of both non-cirrhotic and cirrhotic patients.

**Method:**

We retrospectively studied 175 patients with chronic hepatitis D (CHD) who were followed for at least 6 months (median of 6.3 (0.6–23.6) years). In addition, we selected 175 patients with HBV monoinfection (CHB) who were matched for gender, age, region of origin, HBeAg status, and bilirubin. Liver-related clinical end points were defined as hepatic decompensation (ascites, encephalopathy, variceal bleeding), liver transplantation, HCC, or liver-related death.

**Results:**

Clinical complications developed earlier (4.6 vs. 6.2 years) and more frequently (35.4% vs. 12.6%, *p* < 0.01) in CHD patients. In a multivariate Cox regression, HDV infection was independently associated with the development of end points (*p* < 0.01; HR: 3.0; 95% CI 1.4–6.4). However, in cirrhotic patients there were no significant differences between HBV and HDV in the development of end points. Besides, CHB patients with cirrhosis developed more frequently HCC (35.5%) than CHD patients with cirrhosis (18.5%).

**Conclusion:**

Our results confirmed that HDV leads to a faster progression to cirrhosis compared to HBV. However, once cirrhosis is present, not HDV but the underlying cirrhosis is the dominate intrinsic risk factor for the development of liver-related end points and for the progression to HCC.

**Supplementary Information:**

The online version contains supplementary material available at 10.1007/s12072-023-10575-0.

## Introduction

The hepatitis delta virus (HDV) is a defective RNA virus, which requires the hepatitis B surface antigen (HBsAg) of the hepatitis B virus for assembly virions [1]. Thus, HDV infects only patients with positive HBsAg. Worldwide, approximately 248–291 million individuals were HBsAg positive with a global prevalence of 3.2–3.9% [2]. In a recent analysis, the HDV prevalence in the HBsAg-positive population was approximately 4.5%, with an estimated 9–19 million anti-HDV-positive patients worldwide [3]. HDV is the most severe form of viral hepatitis, which causes an accelerated progression to liver cirrhosis and liver-related clinical end points such as decompensation, liver transplantation, HCC, and death [4–9] compared to HBV monoinfected patients [10, 11]. Progression rates of cirrhosis has been reported to be 62–67%, resulting in HCC rates of 23% [4, 5, 9, 12]. However, all published studies did not distinguish between cirrhotic and non-cirrhotic patients. Besides, whether HDV is a carcinogenic agent and associated with further risk in the increase for HCC development remains controversial [4, 9, 10, 13, 14], whereas the increased risk of HCC in CHB patients and the carcinogen effect of HBV have been well established [15]. In HBV monoinfection, prolonged suppression of HBV replication by NA has been associated with reduced frequency of decompensation and HCC [15]. On the other hand, studies have described an even higher risk for development of liver-related clinical end points in CHD patients receiving therapy with NA [16, 17]. Thus, NA are not recommended in non-cirrhotic CHD patients in the absence of HIV. Nevertheless, NA should be used if criteria for HBV treatment are fulfilled [15]. Besides, therapy with pegylated interferon alpha (PEG-IFNα) with consecutive HDV RNA decline was associated with a more benign clinical long-term outcome [4, 17, 18].

The aim of this study was to evaluate the clinical long-term outcome of CHD patients compared to matched patients monoinfected with HBV in the so far largest European, single-center cohort. In particular, it should be investigated whether the differences in the long-term clinical course of patients with CHB and CHD are confirmed in both non-cirrhotic and cirrhotic patients and whether the treatment strategies have an impact on the long-term course. One further focus should be on the development of HCC and how HDV differs from HBV.

## Methods

### Patients

All anti-HDV-positive patients followed at Hannover Medical School as part of routine clinical follow-up were screened. Baseline was defined as the first visit at the institution. Patients were included in the study if they had detectable HBsAg and either anti-HDV IgM antibodies or HDV RNA for at least 6 months. Only patients with an available follow-up of at least 6 months with a minimum of two visits were included. Patients were excluded if they had undergone liver transplantation or suffered from HCC before the first observation. They were also excluded if they had liver disease caused by non-alcoholic steatohepatitis, alcohol, or metabolic disease. Virological parameters for hepatitis B and delta were measured as previously described. One hundred and thirty-six patients were part of a previous study [17]. According to these inclusion criteria, patients with HBV monoinfection from a large well-defined cohort of hepatitis B patients were screened and matched by gender, age, region of origin, HBeAg status, and bilirubin. For nine patients infected with HDV, data for region of origin were not available. These patients were not matched by region of origin. Additionally, HBeAg at baseline was missing for 24 CHD patients. In this case, the first available HBeAg during follow-up was used.

Liver-related end points were defined as hepatic decompensation (ascites, encephalopathy, variceal bleeding), liver transplantation, HCC, or liver-related death. Cirrhosis was diagnosed based on liver histology (F5 and F6 according to the ISHAK score). If data were not available, cirrhosis was assessed based on transient elastography (≥ 14.0 kPa) [19] or sonographic parameters (i.e., nodular liver) with additionally one or more signs of portal hypertension (platelets < 150,000/mL, collaterals, splenomegaly (largest dimension > 12 cm), and varices).

### Statistics

Statistical analyses and the creation of figures were performed by using SPSS software (SPSS Inc., Chicago, IL, version 28 including the Python-Plug-in for propensity score matching). All parameters were described as mean ± SD. *p* values < 0.05 were considered as statistically significant. Continuous variables were analyzed by *t* tests. For nonparametric parameters Mann–Whitney *U* tests were used. A Chi-square test was calculated for the comparison of discrete variables. In case of an expected cell count ≤ 5, Fisher’s exact test was used instead. Parameters that were associated with the clinical long-term outcome in univariate time-depending COX-regression models (*p* < 0.05) were additionally compared in multivariate analysis. Multivariate COX-regression analyses were performed by using the likelihood ratio test for backward selection. Hazard ratios (HRs), including their 95% confidence intervals (CIs), were calculated for the COX-regression models. Using Kaplan–Meier analysis, we estimated the cumulative event-free survival and compared different risk groups by log-rank tests. To ensure a comprehensible matching of the groups, a propensity score was estimated from a multivariate logistic regression model, containing the above-mentioned matching variables. The subsequent individually matched propensity score was used for nearest neighbor matching of both the groups with caliper match tolerance of 0.2. Due to the low number of patients with CHB in the group of cirrhotic patients, we performed an inverse probability of treatment weighting (IPTW) in this group with subsequent calculation of the outcome using generalized estimating equations and logistic regression model.

## Results

### Baseline characteristics

A cohort of 350 patients was studied with a mean follow-up of 6.9 years (0.60–23.6 years). Of these patients, 175 were infected with HDV with a mean age of 38.5 years (16.0–67.9), 68.6% were male, and most of the patients were born in Eastern Europe (36.6%). The incidence of cirrhosis at the first visit was higher in patients with HDV infection (45.1%) than in HBV monoinfection (17.7%) (*p* < 0.01) (Table 1).

**Table 1:** Baseline characteristics and factors univariately differentiating between CHD and CHB based on ANOVA (continuous values) and Chi-square analysis (discrete values)

	Total cohort = 350	CHD = 175	CHB = 175	*p*-value
Sex (%)	Male = 240 (68.6%)	Male = 120 (68.6%)	Male = 120 (68.6%)	0.55
	Female = 110 (31.4%)	Female = 55 (31.4%)	Female = 55 (31.4%)	
Age, years, mean ± SD (range)	37.6 ± 11.9 (15.0–67.9)	38.5 ± 12.1 (16.0–67.9)	36.8 ± 11.7 (15.0–64.4)	0.17
Country of origin (%)	Central Europe = 73 (20.9%)	Central Europe = 36 (20.6%)	Central Europe = 37 (21.1%)	0.24
	Eastern Mediterranean = 123 (35.1%)	Eastern Mediterranean = 60 (34.3%)	Eastern Mediterranean = 63 (36.0%)	
	Eastern Europe = 128 (36.6%)	Eastern Europe = 64 (36.6%)	Eastern Europe = 64 (36.6%)	
	Other = 16 (4.6%)	Other = 6 (3.4%)	Other = 10 (5.7%)	
AST, mean ± SD (range)	91.2 ± 210.9 (30–2827)	86.6 ± 91.6 (30–691)	70.2 ± 166.5 (30–1390)	0.04
ALT, mean ± SD (range)	122.1 ± 216.9 (31–2507)	113.6 ± 143.9 (31–1238)	67.1 ± 245.9 (32–2507)	0.41
AP, mean ± SD (range)	113.9 ± 75.4 (9–616)	117.5 ± 66.4 (9–512)	105.1 ± 81.7 (25–616)	0.13
gGT, mean ± SD (range)	75.1 ± 122.9 (5–1135)	76.1 ± 84.1 (5–452)	60.7 ± 123.8 (6–976)	0.03
Bilirubin, mean ± SD (range)	18.9 ± 38.6 (5–478)	16.5 ± 20.3 (5–148)	18.3 ± 37.2 (5–348)	0.32
Albumin, mean ± SD (range)	39.9 ± 6.9 (15–70)	38.5 ± 5.9 (22–50)	40.9 ± 7.4 (15–70)	< 0.01
Platelets, mean ± SD (range)	168.8 ± 80.4 (16–450)	139.3 ± 74.7(16–377)	194.8 ± 70.9 (23–398)	< 0.01
INR, mean ± SD (range)	1.15 ± 0.24 (1–3)	1.21 ± 0.24 (1–3)	1.10 ± 0.17 (1–2)	< 0.01
MELD, mean ± SD (range)	8.61 ± 3.03 (3.93 –25.4)	9.52 ± 3.08 (5.4–21.1)	8.10 ± 2.81 (5.24–25.4)	< 0.01
Log HBV DNA mean ± SD (range)	3.35 ± 2.41 (0–9.04)	2.13 ± 1.89 (0–8.04)	4.23 ± 2.36 (0–9.04)	< 0.01
HBV DNA neg	65 (18.5%)	43 (28.1%)	13 (8.7%)	< 0.01
antiHBe pos	239 (79.4%)	121 (81.8%)	118 (77.1%)	0.32
HBeAg positive, *n* (%)	68 (19.4%)	29 (15.7%)	39 (22.3%)	0.59
Cirrhosis, *n* patients (%)	110 (31.4%)	79 (45.1%)	31 (17.7%)	< 0.01
Biopsy, *n* patients, fibrosis stage mean ± SD (range)	174, 2.22 ± 1.81 (0–6)	82, 3.45 ± 1.56 (0–6)	92, 2.12 ± 1.77 (0–6)	< 0.01
Fibroscan (kPa) mean ± SD (range)	11.4 ± 10.17 (2.9–50.1)	13.9 ± 11.12 (3.4–50.1)	6.4 ± 5.24 (2.9–28.4)	< 0.01
APRI, mean ± SD (range)	2.12 ± 6.21 (0.1–33)	3.32 ± 8.91 (0.2–33)	1.18 ± 2.28 (0.1–20.9)	< 0.01
FIB-4, mean ± SD (range)	2.70 ± 3.51 (0.1–34.7)	3.87 ± 4.36 (0.17–34.7)	1.58 ± 1.82 (0.10–11.5)	< 0.01
Antiviral therapy (%)	No therapy = 111 (31.7%)	No therapy = 44 (25.1%)	No therapy = 67 (38.3%)	< 0.01
	NA = 152 (43.4%)	NA = 64 (36.6%)	NA = 88 (50.3%)	
	IFN = 87 (24.9%)	IFN = 67 (38.3%)	IFN = 20 (11.4%)	
Follow-up, mean ± SD (range)	6.93 ± 5.12 (0.60–23.6)	6.29 ± 4.97 (0.60–23.6)	7.57 ± 5.22 (0.60–22.9)	0.02

### Clinical long-term outcome

**Fig. 1:**
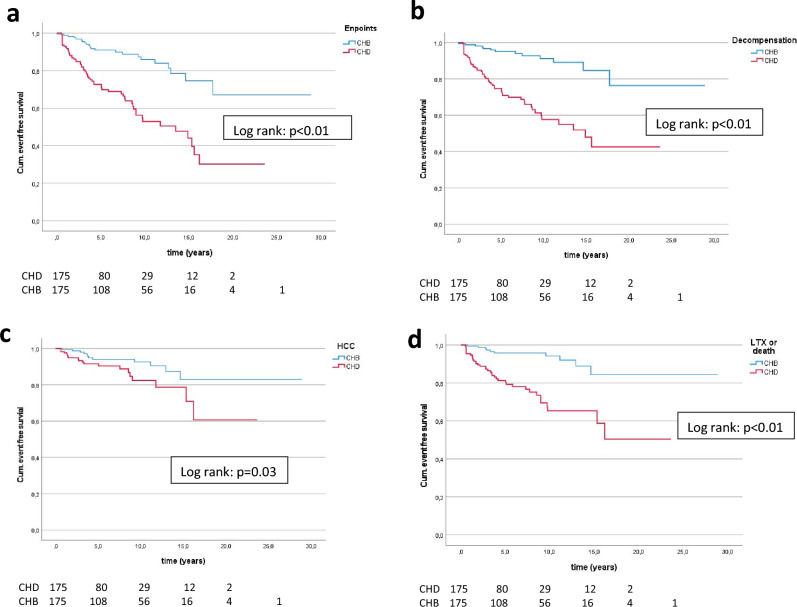
Cumulative event-free survival in the group of CHD patients, compared with CHB patients. Risk-free survival for overall end points (**a**), decompensation (**b**), HCC (**c**), death and/or LTX (**d**)

During follow-up, 29 patients progressed to cirrhosis within a mean time of 3.31 years (0.60–12.0 years), and all of them were anti-HDV positive. At least one liver-related clinical end point was observed in 84 patients; in 35.4% of the CHD patients after a mean follow-up of 4.6 years (0.50–16.2) and in 12.6% of the CHB patients after 6.18 years (0.50–17.7) (*p* < 0.01). Decompensation occurred in 68 patients (31.4% CHD, 7.4% CHB) (*p* < 0.01). Liver transplantation and/or death was observed in 22.3% of the patients with HDV infection and in 5.7% with HBV infection (*p* < 0.01). Fourteen patients died (10 with CHD, 4 with CHB) and 37 underwent liver transplantation (30 with CHD, 7 with CHB). HCC (*n* = 32) was detected in 11.4% of patients with CHD and in 6.9% of patients with CHB (*p* = 0.1). End points developed earlier in patients infected with HDV (decompensation: 4 years vs. 6 years; LTX and/or death: 4 years vs. 6.4 years; HCC: 4 years vs. 6 years). The poor clinical outcome of CHD patients compared to CHB patients was confirmed in Kaplan–Meier analysis (log rank: *p* < 0.01) (Fig. 1 a-d). Of note, clinical end points in CHD patients were only developed with present cirrhosis (at baseline or during follow-up). Besides, in CHB patients, two patients developed liver-related end points without cirrhosis (1 HCC, 1 decompensation).

For better comprehensibility of the matching, we also performed a propensity score matching (CHB: 129 vs. CHD: 129), which confirmed the worse course of hepatitis delta in both the Chi-square analysis analysis (*p* < 0.01) and the Kaplan–Meier analysis (log rank; *p* < 0.01) (supplement figure).

### Patients with cirrhosis

**Fig. 2:**
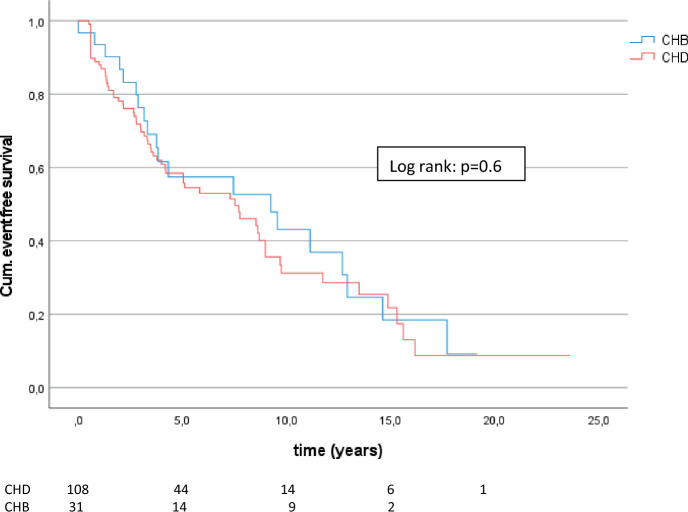
Cumulative event-free survival of CHD with cirrhosis compared with CHB patients with cirrhosis

Interestingly, there were no differences in the development of liver-related clinical end points, decompensation, and liver transplantation and/or death considering patients with cirrhosis only (*n* = 139; 64.5% HBV, 57.4% HDV) in Chi-square analysis. Besides, CHB patients with cirrhosis developed more often HCC (35.5%) than CHD patients with cirrhosis (18.5%) (*p* = 0.04). Kaplan–Meier analysis confirmed that there were no significant differences between HBV and HDV in the development of end points (log rank *p* = 0.6) (Fig. 2), decompensation (log rank *p* = 0.1), LTX, and/or death (log rank *p* = 0.3). Even the development of HCC was not associated with HDV in the time-depending model (log rank *p* = 0.3).

In this group, we also wanted to provide an exact comparison of the two groups. Due to the unequal distribution of the number of patients (CHB: 31 vs. CHD: 108), the inverse probability of treatment weighting was performed. The subsequent logistic regression showed no differences in the development of end points between CHB and CHD (*p* = 0.95; OR: 1.03; 95% CI 0.41–2.56).

### Antiviral therapy and liver-related end points

**Fig. 3:**
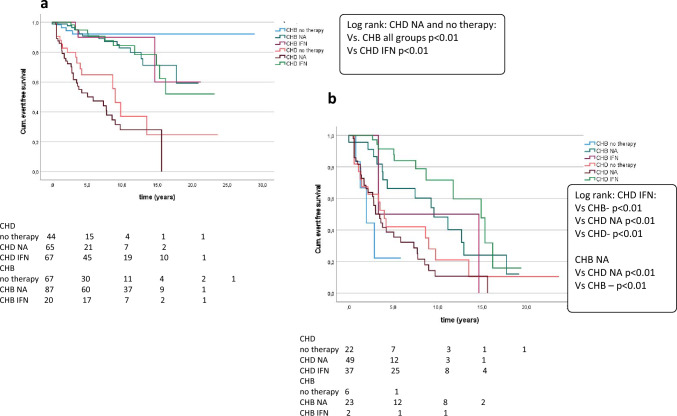
Cumulative event-free survival of CHD with different treatment (IFN, NA, no therapy) compared with CHB patients with different treatment (IFN, NA, no therapy). Risk-free survival for non-cirrhotic (**a**) and cirrhotic patients (**b**). Log rank was described for significant analysis only

Treatment administration is shown in Table 1. CHD patients developed liver-related clinical end points most frequently with no therapy or NA compared to all other treatment groups in Kaplan–Meier analysis (*p* < 0.01). In contrast, patients with HDV infection and IFN treatment had a favorable clinical long-term follow-up (*p* < 0.01), equal to patients with HBV monoinfection (Fig. 3a).

These data were confirmed in cirrhotic patients. Besides, patients with HBV monoinfection who were treated with NA had a more benign clinical long-term follow-up compared to CHB patients without therapy (*p* < 0.01) (Fig. 3b). Of note, every CHB patients with cirrhosis had negative HBV DNA at the end of follow-up or were at therapy with NA.

### Factors associated with the development of clinical end points

We next investigated which factors correlated with clinical end points in uni- and multivariate Cox-regression analysis for all patients (*n* = 350) (Table 2a). The analysis of virological parameters can be found in the supplement. Of note, biochemical disease activity, as determined by AST (*p* = 0.12; CI 1.00–1.00) and ALT levels (*p* = 0.73; CI 1.00–1.00), or HBV DNA (*p* = 0.44; CI 1.00–1.00), was not associated with the clinical long-term outcome in cirrhotic or non-cirrhotic patients. In univariate COX-regression model, therapy with IFN was linked to a more benign clinical outcome (*p* < 0.01; HR: 0.51; 95% CI 0.32–0.83), whereas treatment with NA was associated with the development of end points (*p* < 0.01; HR: 1.92; 95% CI 1.18–2.91). Multivariate backward COX-regression model analysis revealed that HDV infection among others was an independent factor associated with the development of end points (*p* < 0.01; HR: 3.03; 95% CI 1.42–6.39). It is important to know that age was also independently associated with the clinical long-term outcome (*p* < 0.01; HR: 1.10; 95% CI 1.00–1.13).

**Table 2:** a,b Parameters associated with the clinical long-term outcome in univariate and multivariate analysis Cox-regression model—(all patients), (patients with cirrhosis)

Parameter	Significance (univariate)	Significance (multivariate)
a Parameters associated with the clinical long-term outcome in univariate and multivariate analysis Cox-regression model (all patients)		
HDV pos	*p* < 0.01; HR: 3.58; 95% CI 2.22–5.92	*p* < 0.01; HR: 3.03; 95% CI 1.42–6.39
Age (linear)	*p* < 0.01; HR: 1.10; 95% CI 1.04–1.08	*p* < 0.01; HR: 1.10; 95% CI 1.00–1.13
Gender (male)	*p* = 0.04; HR: 1.77; 95% CI 1.11–2.89	*p* = 0.05; HR: 2.12; 95% CI 1.00–4.54
Cirrhosis	*p* < 0.01; HR: 11.6; 95% CI 6.82–19.7	Not significant
gGT (linear)	*p* < 0.01; HR: 1.00; 95% CI 1.00–1.01	*p* < 0.01; HR: 1.00; 95% CI 1.00–1.10
Alcalic phosphatase (linear)	*p* = 0.01; HR: 1.00; 95% CI 1.00–1.01	*p* < 0.01; HR: 1.00; 95% CI 1.00–1.10
Bilirubin (linear)	*p* < 0.01; HR: 1.00; 95% CI 1.00–1.01	Not significant
Albumin (linear)	*p* < 0.01; HR: 0.87; 95% CI 0.81–0.93	*p* < 0.01; HR: 0.93; 95% CI 0.82–0.94
Platelets (linear)	*p* < 0.01; HR: 0.98; 95% CI 0.98–0.99	*p* < 0.01; HR: 0.98; 95% CI 0.98–0.99
INR	*p* < 0.01; HR: 55.5; 95% CI 27.4–112.1	*p* < 0.01; HR: 9.93; 95% CI 2.24–46.2
HBeAg negative	*p* < 0.01; HR: 3.49; 95% CI 1.61–7.58	Not significant
Therapy (NA)	*p* < 0.01; HR: 1.92; 95% CI 1.18–2.91	Not significant
Therapy (IFN)	*p* < 0.01; HR: 0.51; 95% CI 0.32–0.83	Not significant
b Parameters associated with the clinical long-term outcome in univariate and multivariate analysis Cox-regression model (patients with cirrhosis)		
Age (linear)	*p* < 0.01; HR: 1.03; 95% CI 1.01–1.06	Not significant
gGT (linear)	*p* < 0.01; HR: 1.00; 95% CI 1.00–1.01	*p* = 0.02; HR: 1.03; 95% CI 1.00–1.01
Alcalic phosphatase (linear)	*p* = 0.01; HR: 1.01; 95% CI 1.01–1.02	*p* < 0.01; HR: 1.01; 95% CI 1.00–1.02
Bilirubin (linear)	*p* < 0.01; HR: 1.01; 95% CI 1.01–1.02	Not significant
Albumin (linear)	*p* < 0.01; HR: 0.89; 95% CI 0.86–0.93	*p* = 0.02; HR: 0.93; 95% CI 0.82–0.91
Platelets (linear)	*p* < 0.01; HR: 0.99; 95% CI 0.98–0.99	*p* = 0.02; HR: 0.99; 95% CI 0.98–0.99
INR	*p* < 0.01; HR: 14.6; 95% CI 6.07–34.9	*p* = 0.04; HR: 4.68; 95% CI 1.12–19.59
Therapy (IFN)	*p* < 0.01; HR: 0.32; 95% CI 0.17–0.57	Not significant
Therapy (NA)	*p* = 0.02; HR: 1.72; 95% CI 1.11–2.68	Not significant

In patients with cirrhosis, HDV infection was not associated with the long-term outcome (*p* = 0.61; CI 0.69–1.89), whereas therapy with IFN and therapy with NA was univariably associated with the clinical outcome (*p* < 0.01; CI 0.17–0.58), (*p* = 0.02; CI 1.11–2.68) (Table 2b).

## Discussion

Hepatitis Delta is the most severe form of viral hepatitis, which was confirmed in multiple studies. However, previous studies comparing the clinical course of HDV and HBV infection did not compare matched patients, have not distinguished between non-cirrhotic and cirrhotic patients, and had a smaller sample size of CHD patients. In our so far largest European single-center cohort study, we could confirm previous studies that patients with HDV infection had a progressive clinical course toward cirrhosis and liver-related end points compared to patients monoinfected with HBV [4–7, 9]. Importantly, HDV infection was an independent factor for a worse clinical long-term outcome in the overall cohort. We primarily assign the development of end points to the rapid and high progression rate toward cirrhosis in CHD patients (18% in CHB vs. 62% in CHD), which has also been shown in previous studies [5], rather than HDV per se. We draw these conclusions from the finding that, in CHD patients, only those with cirrhosis or advanced liver fibrosis at baseline, who progressed to liver cirrhosis during follow-up, developed complications. Besides, age was an independent factor in multivariate analyses for a worse clinical long-term outcome in the total cohort. However, if patients progressed to cirrhosis, age was only univariatly associated with a poor clinical outcome. Previous studies also showed that CHD patients were younger than CHB patients [4, 7, 10]. Thus, it can be deduced that HDV induces a fast progression to cirrhosis in younger age, leading to prolonged cumulative risk for the consecutive development of end points. However, once patient developed liver cirrhosis, all patients had a worse clinical long-term outcome regardless of whether they were infected with HDV or HBV. Therefore, we hypothesize that cirrhosis is the dominant risk factor for the development of liver-related clinical complication without being influenced by additional other intrinsic factors such as HDV. This is in line with one recent study, where the occurrence of total events did not differ between HBV and HDV after propensity score matching by age and frequency of cirrhosis [14]. These results suggested that HDV itself may contribute to liver damage and consecutive cirrhosis, which can be implied by correlating plasma levels of HDV RNA [20] and different inflammatory profiles compared to HBV [21]. But, if cirrhosis was present, positive HDV RNA (qualitative) did not correlate anymore with outcome, hypothesizing that HDV itself causes no further damage in cirrhotic patients, which was confirmed in our study. However, the mechanism by which HDV causes the progression has not been identified and has to be investigated in further studies. Besides, in the context of our data, it should also be questioned whether the reduction or absence of HDV RNA is a sufficient therapeutic outcome, especially in cirrhotic patients. To confirm our data, more attention should be paid to the distinction between cirrhotic and non-cirrhotic patients in further clinical trials.

One further focus was the evaluation of the progression to HCC. CHB patients with cirrhosis developed more frequently HCC (35.5%) than CHD patients with cirrhosis (18.5%) (*p* = 0.04). In contrast, one systematic review reported an increased risk for the development of HCC in patients with HDV infection with a pooled odds ratio of 1.28 compared to patients with HBV monoinfection, but this association could be confirmed in studies published in Asia only [12]. In our analysis, only four patients with HBV monoinfection and four patients with HDV infection were born in an Asian country. Another systematic review with only longitudinal cohort studies reported that CHD patients were at twofold increased risk of developing HCC compared to CHB patients, but comparing patients with cirrhosis only, the HCC risk was not statistically different, although the included studies were difficult to compare [13]. In our analysis, HCC only developed in the presence of cirrhosis in CHD patients, whereas one patient monoinfected with HBV who developed HCC had no cirrhosis at all. Thus, this analysis may be indicating that the progression to HCC is not associated with HDV, but that HBV and the frequent progression to cirrhosis may play a role in the pathogenesis. Although previous data indicated that HDV can induce pathways related to fibrosis and hepatocarcinogenesis such as TGF-β, NF-κB and JAK-STAT, a direct oncogenic effect of HDV is doubtful since this virus does not integrate into the human genome [21]. Thus, further evaluation of the pathogenesis of HDV is urgently needed. Besides, Farci et al. suggested lower HCC rates in HDV-infected patients treated with IFN compared to untreated patients, indicating that IFN may have a protective effect [22]. However in our study, IFN alone was not associated with HCC, challenging the hypotheses of IFN as HCC-protective agent, like other studies [23]. But, CHD patients who were treated with IFN-based therapy had a more benign clinical long-term outcome. Most important, even in patients with cirrhosis, previous IFN-based therapy was linked to a better clinical outcome in univariate analysis, which is in line with findings in patients with the HCV infection achieving suppression of viral replication by antiviral therapy [24]. However, treatment with NA was associated with an even more severe clinical outcome in our cohort with CHD patients and in previous data [4, 16–18], challenging the use of NA in CHD patients.

Several limitations of this study need to be considered. First of all, the diagnosis of cirrhosis (*n* = 110) was based on the gold standard of histopathology in 60 patients only. However, patients who were suspected of having cirrhosis by fibroscan (*n* = 17) or sonography (*n* = 33) were re-examined. Importantly, they showed one or more signs of portal hypertension (see methods). Thus, the diagnosis of cirrhosis was confirmed by extensive clinical evaluation. Besides, although we had a large database of 350 patients, the numbers of patients in special subgroups were limited. Moreover, based on the retrospective character of the study, few parameters were missing, especially quantitative HBV DNA and HDV RNA. This disabled us from studying quantitative HDV RNA levels given that this information was available only for a limited number of cases and the quantitative HDV RNA assays have changed over the entire observation period, making it impossible to compare the available HDV RNA values. Additionally, serum samples for retesting of virological parameters with improved assays were not always available, and storage conditions and time might have influenced test results. Moreover, although patients were matched by gender, age, region of origin, HBeAg status and bilirubin, patients with HBV monoinfection had less progressive disease indicated by albumin, INR, and platelet count and had less frequent cirrhosis. However, there were no differences in this parameter, once cirrhosis was present. That is why we additionally performed propensity score matching and inverse probability of treatment weighting. Furthermore, as previously discussed, the benign clinical long-term outcome associated with IFN therapy in CHD patients may be biased by the administration to patients with compensated liver disease only [17]. Besides, the clinical long-term outcomes of the new treatment strategies, in particular the effect of the progression rate to cirrhosis, are of special interest [25].

In summary, our study showed that HDV infection is an independent factor of disease progression, especially for the development of cirrhosis in younger age. Once patients progressed to cirrhosis, HBV and HDV infection had a similar clinical long-term outcome. This suggests that cirrhosis, rather than HDV, is the dominant risk factor for the development of end points. Further studies are needed to analyze the impact of HDV on the development of HCC, as we found no risk for the increase of HCC development in cirrhotic patients with CHD compared to CHB patients. Moreover, patients infected with HDV and treated with IFN developed less clinical events and the long-term outcome did not differ in patients monoinfected with HBV.

### Supplementary Information

Below is the link to the electronic supplementary material.

Supplementary file1 (EPS 1305 KB)

Supplementary file2 (EPS 1179 KB)

Supplementary file3 (TIF 47 KB)

Supplementary file4 (DOCX 13 KB)

## Data Availability

The data that support the findings of this study are available on reasonable request from Anika Wranke.

## References

[1] Urban S, Neumann-Haefelin C, Lampertico P (2021). Hepatitis D virus in 2021: virology, immunology and new treatment approaches for a difficult-to-treat disease. Gut.

[2] Polaris Observatory Collaborators (2018). Global prevalence, treatment, and prevention of hepatitis B virus infection in 2016: a modelling study. Lancet Gastroenterol Hepatol.

[3] Stockdale AJ, Kreuels B, Henrion MYR, Giorgi E, Kyomuhangi I, de Martel C (2020). The global prevalence of hepatitis D virus infection: Systematic review and meta-analysis. J Hepatol.

[4] Niro GA, Smedile A, Ippolito AM, Ciancio A, Fontana R, Olivero A (2010). Outcome of chronic delta hepatitis in Italy: a long-term cohort study. J Hepatol.

[5] Buti M, Homs M, Rodriguez-Frias F, Funalleras G, Jardi R, Sauleda S (2011). Clinical outcome of acute and chronic hepatitis delta over time: a long-term follow-up study. J Viral Hepat.

[6] Braga WS, de Oliveira CM, de Araujo JR, Castilho Mda C, Rocha JM, Gimaque JB (2014). Chronic HDV/HBV co-infection: predictors of disease stage–a case series of HDV-3 patients. J Hepatol.

[7] Kamal H, Westman G, Falconer K, Duberg A, Weiland O, Haverinen S (2020). Long-term study of hepatitis Delta virus infection at secondary care centers: the impact of viremia on liver-related outcomes. Hepatology.

[8] Roulot D, Brichler S, Layese R, BenAbdesselam Z, Zoulim F, Thibault V, Deltavir study group (2020). Origin, HDV genotype and persistent viremia determine outcome and treatment response in patients with chronic hepatitis delta. J Hepatol.

[9] Romeo R, Del Ninno E, Rumi M, Russo A, Sangiovanni A, de Franchis R (2009). A 28-year study of the course of hepatitis Delta infection: a risk factor for cirrhosis and hepatocellular carcinoma. Gastroenterology.

[10] Fattovich G, Giustina G, Christensen E, Pantalena M, Zagni I, Realdi G (2000). Influence of hepatitis delta virus infection on morbidity and mortality in compensated cirrhosis type B. The European concerted action on viral hepatitis (Eurohep). Gut.

[11] Brancaccio G, Fasano M, Grossi A, Santantonio TA, Gaeta GB (2019). Clinical outcomes in patients with hepatitis D, cirrhosis and persistent hepatitis B virus replication, and receiving long-term tenofovir or entecavir. Aliment Pharmacol Ther.

[12] Alfaiate D, Clément S, Gomes D, Goossens N, Negro F (2020). Chronic hepatitis D and hepatocellular carcinoma: a systematic review and meta-analysis of observational studies. J Hepatol.

[13] Kamal H, Fornes R, Simin J, Stål P, Duberg A, Brusselaers N (2021). Risk of hepatocellular carcinoma in hepatitis B and D virus co-infected patients: a systematic review and meta-analysis of longitudinal studies. J Viral Hepat.

[14] Bockmann JH, Grube M, Hamed V, von Felden J, Landahl J, Wehmeyer M (2020). High rates of cirrhosis and severe clinical events in patients with HBV/HDV co-infection: longitudinal analysis of a German cohort. BMC Gastroenterol.

[15] Cornberg M, Lok AS, Terrault NA, Zoulim F, 2019 EASL-AASLD HBV Treatment Endpoints Conference Faculty (2019). Guidance for design and endpoints of clinical trials in chronic hepatitis B - Report from the 2019 EASL-AASLD HBV treatment endpoints conference. Hepatology.

[16] Scheller L, Hilgard G, Anastasiou O, Dittmer U, Kahraman A, Wedemeyer H (2021). Poor clinical and virological outcome of nucleos(t)ide analogue monotherapy in HBV/HDV co-infected patients. Medicine (Baltimore).

[17] Wranke A, Serrano BC, Heidrich B, Kirschner J, Bremer B, Lehmann P (2017). Antiviral treatment and liver-related complications in hepatitis delta. Hepatology.

[18] Yurdaydin C, Keskin O, Kalkan C, Karakaya F, Caliskan A, Kabacam G (2018). Interferon treatment duration in patients with chronic delta hepatitis and its effect on the natural course of the disease. J Infect Dis.

[19] Da BL, Surana P, Takyar V, Kleiner DE, Heller T, Koh C (2020). Vibration-controlled transient elastography for the detection of cirrhosis in chronic hepatitis D infection. J Viral Hepat.

[20] Romeo R, Foglieni B, Casazza G, Spreafico M, Colombo M, Prati D (2014). High serum levels of HDV RNA are predictors of cirrhosis and liver cancer in patients with chronic hepatitis delta. PLoS ONE.

[21] Puigvehí M, Moctezuma-Velázquez C, Villanueva A, Llovet JM (2019). The oncogenic role of hepatitis delta virus in hepatocellular carcinoma. JHEP Rep.

[22] Farci P, Roskams T, Chessa L, Peddis G, Mazzoleni AP, Scioscia R (2004). Long-term benefit of interferon alpha therapy of chronic hepatitis D: regression of advanced hepatic fibrosis. Gastroenterology.

[23] Su CW, Huang YH, Huo TI, Shih HH, Sheen IJ, Chen SW (2006). Genotypes and viremia of hepatitis B and D viruses are associated with outcomes of chronic hepatitis D patients. Gastroenterology.

[24] van der Meer AJ, Wedemeyer H, Feld JJ, Dufour JF, Zeuzem S, Hansen BE (2014). Life expectancy in patients with chronic HCV infection and cirrhosis compared with a general population. JAMA.

[25] Rizzetto M, Hamid S, Negro F (2021). The changing context of hepatitis D. J Hepatol.

