# Supplementary figures and images for: Clinical long-term outcome of hepatitis D compared to hepatitis B monoinfection
Source: Hepatol Int. 2023 Oct 3;17(6):1359–67. doi: 10.1007/s12072-023-10575-0 (PMC10661878; doi:10.1007/s12072-023-10575-0)

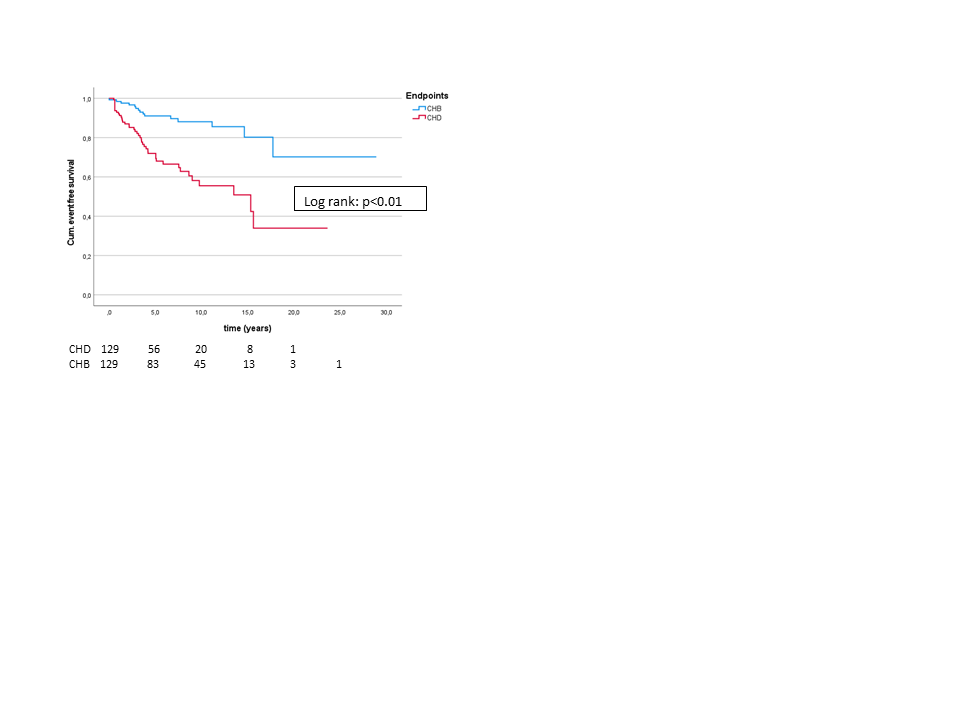

Supplement: Supplementary file 3 — Supplementary file3 (TIF 47 KB) [file 12072_2023_10575_MOESM3_ESM.tif]
