# Supplementary material for: Clinical long-term outcome of hepatitis D compared to hepatitis B monoinfection
Source: Hepatol Int. 2023 Oct 3;17(6):1359–67. doi: 10.1007/s12072-023-10575-0 (PMC10661878; doi:10.1007/s12072-023-10575-0)
Supplement: Supplementary file 4 — Supplementary file4 (DOCX 13 KB) [file 12072_2023_10575_MOESM4_ESM.docx]

**Supplement**

In Kaplan Meier analysis CHD patients with positive antiHBe, negative HBeAg and negative HBV DNA had the most progressive course and developed endpoints more frequently compared to the other groups (figure 3). On the other hand, CHB patients with negative antiHBe had a favorable clinical outcome (figure 2). In addition, HBV-infected patient with end stage liver disease had significantly higher HBV DNA levels compared to HDV (log HBV DNA 4.40 vs. 1.60 in HDV) (p<0.01). In the total cohort of CHD patients positive HDV RNA at baseline or at end of follow up were associated with the development of clinical endpoints (p=0.01 and p<0.01). However, in the group of cirrhotic CHD patients HDV RNA were not linked to a worse clinical outcome (p=0.3, p=0.6).

**Supplement figure legends**

**Suppl. Fig.1** Cumulative event-free survival in the group of CHD patients, compared with CHB patients after propensity score matching. Risk-free survival for overall endpoints

**Suppl. Fig.2** Cumulative event-free survival of CHD patients compared with CHB patients based on virological factors HBV DNA (A), HBeAg (B) and antiHBe (C). Log rank were described for significant analysis only
